# How Traumatic Experiences Leave Their Signature on the Genome: An Overview of Epigenetic Pathways in PTSD

**DOI:** 10.3389/fpsyt.2014.00093

**Published:** 2014-07-31

**Authors:** Tania L. Roth

**Affiliations:** ^1^Department of Psychological and Brain Sciences, University of Delaware, Newark, DE, USA

**Keywords:** DNA methylation, histones, miRNA, stress, fear, PTSD

Epigenetic mechanisms are a class of molecular mechanisms by which environmental influences, including stress, can interact with the genome to have long-term consequences for brain plasticity and behavior. As PTSD, by definition, requires exposure to a traumatic event, and because genes are exquisitely sensitive to stress and trauma, epigenetic alterations have received attention as possible contributors to the development and persistence of PTSD symptoms. In this research topic, empirical support for the role of epigenetics in PTSD are presented and discussed. The articles assembled here cover a range of disciplines and experimental approaches in both animal models and humans that link stress and trauma with epigenetic alterations. Many articles also offer perspectives on epigenetics and sex differences, diagnosis and intervention, and future directions to bridge the gap between basic and clinical work.

Two original research articles provide empirical support for DNA methylation as a useful biomarker, not only in the detection and diagnosis of PTSD, but also as a biological measure for prediction of response to treatment, monitoring treatment efficacy, and prognosis of outcome. Rusiecki et al. (1) explore changes in DNA methylation of immune-related genes in US military service members with a PTSD diagnosis, showing differential patterns of methylation present pre- vs. post-deployment. Yehuda et al. (2) explore DNA methylation changes in association with changes in PTSD symptoms and other biological measures (including cortisol levels) in responders and non-responders to psychotherapy treatment.

Three review articles describe the compelling evidence for epigenetic alterations, particularly DNA methylation, as a consequence of exposure to stress encountered early in development. Raabe and Spengler (3) discuss studies showing early-life stress induced epigenetic alterations of stress genes as an important pathway in the dysregulation of stress systems in rodents and patients. Karsten and Baram (4) review the neuroanatomical and molecular pathways bridging sensory input with gene expression programing. They especially focus on how either nurturing or aversive early-life experiences can alter regulation of corticotropin-releasing hormone gene expression in hypothalamic neurons. McGowan (5) discusses studies of humans and animal model analogs that address molecular mechanisms underlying changes in stress-sensitive physiological systems in response to early-life trauma, paying particular attention to work on the glucocorticoid receptor. In an original research article, Kundakovic et al. (6) use a rodent model of early-life adversity (separation of infant mice from the mother) to explore the relationship between stress, genetic background, and sex in the determination of neurobehavioral and epigenetic outcomes. Together, data presented in these four articles are consistent with the notion that epigenetic programing early in life confers an enhanced risk on disease development upon re-exposure to trauma or stress. Throughout these articles, sex-specific differences at the epigenetic level are apparent too, suggesting that epigenetic activity plays an important role in sex-specificity and susceptibility to stress.

Continuing with a developmental theme, two review articles provide fascinating perspectives on the relationship between brain development and plasticity, gene × environment interactions, and the development of fear systems. Callaghan et al. (7) discuss developmental transitions in emotional learning and the role early-life stress has in both prematurely closing critical period plasticity and accelerating the development of fear learning systems. They also discuss the provocative idea of reopening critical periods of emotional learning to help treat many anxiety disorders. Nabel and Morishita (8) consider the potential contributions of “molecular brakes” identified in visual system development, the major model of critical period plasticity, to the development of fear system connections. They also discuss epigenetic regulators in the context of fear system development and their potential as new targets for therapeutic intervention.

One of the most common problems associated with PTSD is the persistence of memories of traumatic events. Since decades of research has shown that changes in gene expression occur when a memory is formed and stored, investigators have explored the relationship between DNA methylation and histone modifications and long-term trajectories in gene regulation associated with fear memories. These data are extensively described in two review articles. Maddox et al. (9) review the role of epigenetic mechanisms in animal models of fear learning and memory (Pavlovian fear conditioning paradigms that produce robust and long-lasting fear memories in rodents), highlighting epigenetic modulation of *FKBP5* in animal models of PTSD and clinical populations. Zovkic et al. (10) review literature supporting the involvement of epigenetics in PTSD, discussing data in the broader context of epigenetics in stress and fear learning. They also focus on evidence for epigenetic mechanisms as regulators of predisposition and resilience to PTSD, and provide a technical overview of approaches for measuring DNA methylation to encourage future investigation of epigenetic mechanisms in animal models of PTSD.

Finally, though it is clear throughout this topic that DNA methylation has been the most extensively studied epigenetic alteration in outcomes associated with stress, evidence for histone modifications and microRNAs (miRNAs) as key epigenetic players are also emerging. Reul (11) discusses how psychologically stressful events evoke a long-term impact on behavior through changes in hippocampal function. Data are presented showing that this can occur through glutamatergic and glucocorticoid-driven changes in epigenetic regulation of gene transcription (via histone acetylation for example) within dentate gyrus neurons. In an original research article, Schmidt et al. (12) explore cortical miRNA expression profiles in a rodent model of PTSD. miRNAs are a more recent recognized class of epigenetic modulators of gene activity (or even a regulator of epigenetic processes), and are small non-coding RNAs that can regulate gene expression post-transcriptionally. Selective serotonin reuptake inhibitors (SSRIs) are the only FDA approved treatment for PTSD, with some evidence that one SSRI, fluoxetine, can ameliorate a subset of PTSD symptoms. These authors also examine fluoxetine effects on miRNA profiles, which may provide insight into the mechanisms underlying treatment effects of antidepressants in PTSD.

## Conflict of Interest Statement

The author declares that the research was conducted in the absence of any commercial or financial relationships that could be construed as a potential conflict of interest.
